# Supplementary material for: Spatiotemporal dynamics of clonal selection and diversification in normal endometrial epithelium
Source: Nat Commun. 2022 Feb 17;13:943. doi: 10.1038/s41467-022-28568-2 (PMC8854701; doi:10.1038/s41467-022-28568-2)
Supplement: Supplementary file 1 — Supplementary Information [file 41467_2022_28568_MOESM1_ESM.pdf]

**Title: Spatiotemporal dynamics of clonal selection and diversification in normal endometrial epithelium**

Manako Yamaguchi[1], Hirofumi Nakaoka[2,3], Kazuaki Suda[1], Kosuke Yoshihara[1], Tatsuya Ishiguro[1], Nozomi Yachida[1], Kyota Saito[1], Haruka Ueda[1], Kentaro Sugino[1], Yutaro Mori[1], Kaoru Yamawaki[1], Ryo Tamura[1], Sundaramoorthy Revathidevi[2], Teiichi Motoyama[4], Kazuki Tainaka[5,6], Roel G. W. Verhaak[7,8], Ituro Inoue[2], Takayuki Enomoto[1]

[1] Department of Obstetrics and Gynecology, Niigata University Graduate School of Medical and Dental Sciences, Niigata 951-8510, Japan.

[2] Human Genetics Laboratory, National Institute of Genetics, Mishima 411-8540, Japan.

[3] Department of Cancer Genome Research, Sasaki Institute, Sasaki Foundation, Chiyoda-ku 101-0062, Japan.

[4] Department of Molecular and Diagnostic Pathology, Niigata University Graduate School of Medical and Dental Sciences, Niigata 951-8510, Japan.

[5] Department of System Pathology for Neurological Disorders, Brain Research Institute, Niigata University, Niigata 951-8585, Japan.

[6] Laboratory for Synthetic Biology, RIKEN Center for Biosystems Dynamics Research, Suita 565-5241, Japan.

[7] The Jackson Laboratory for Genomic Medicine, Farmington, CT, USA.

[8] Department of Neurosurgery, Cancer Center Amsterdam, Amsterdam UMC, VU University Medical Center (VUmc), 1081 HV Amsterdam, The Netherlands.

**Supplementary information**

**Supplementary Figures 1-10**
**Supplementary Tables 1-3**

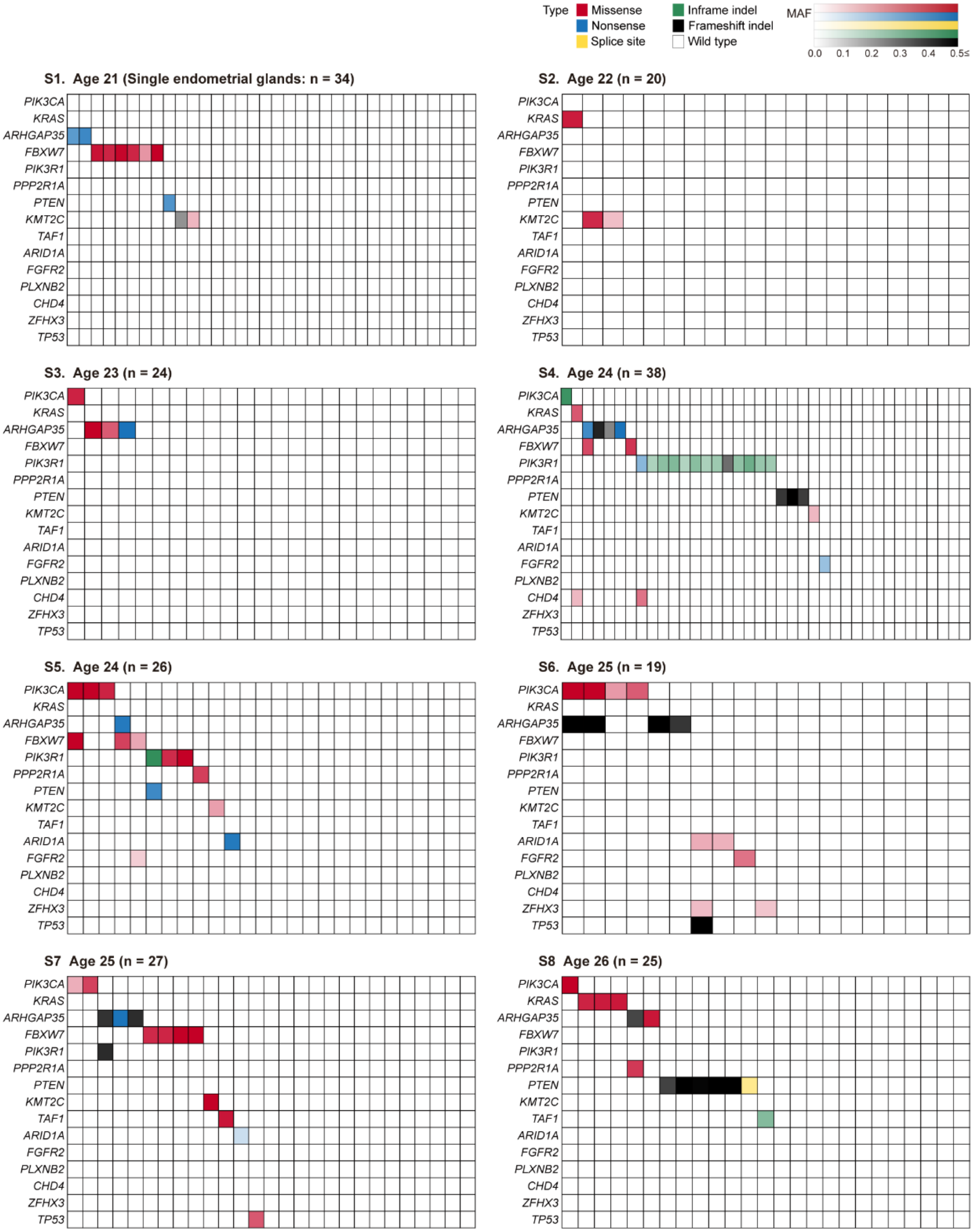

Type
Missense
Nonsense
Splice site
Inframe indel
Frameshift indel
Wild type
MAF
0.0 0.1 0.2 0.3 0.4 0.5≤
S1. Age 21 (Single endometrial glands: n = 34)
S2. Age 22 (n = 20)
S3. Age 23 (n = 24)
S4. Age 24 (n = 38)
S5. Age 24 (n = 26)
S6. Age 25 (n = 19)
S7 Age 25 (n = 27)
S8 Age 26 (n = 25)
PIK3CA
KRAS
ARHGAP35
FBXW7
PIK3R1
PPP2R1A
PTEN
KMT2C
TAF1
ARID1A
FGFR2
PLXNB2
CHD4
ZFHX3
TP53

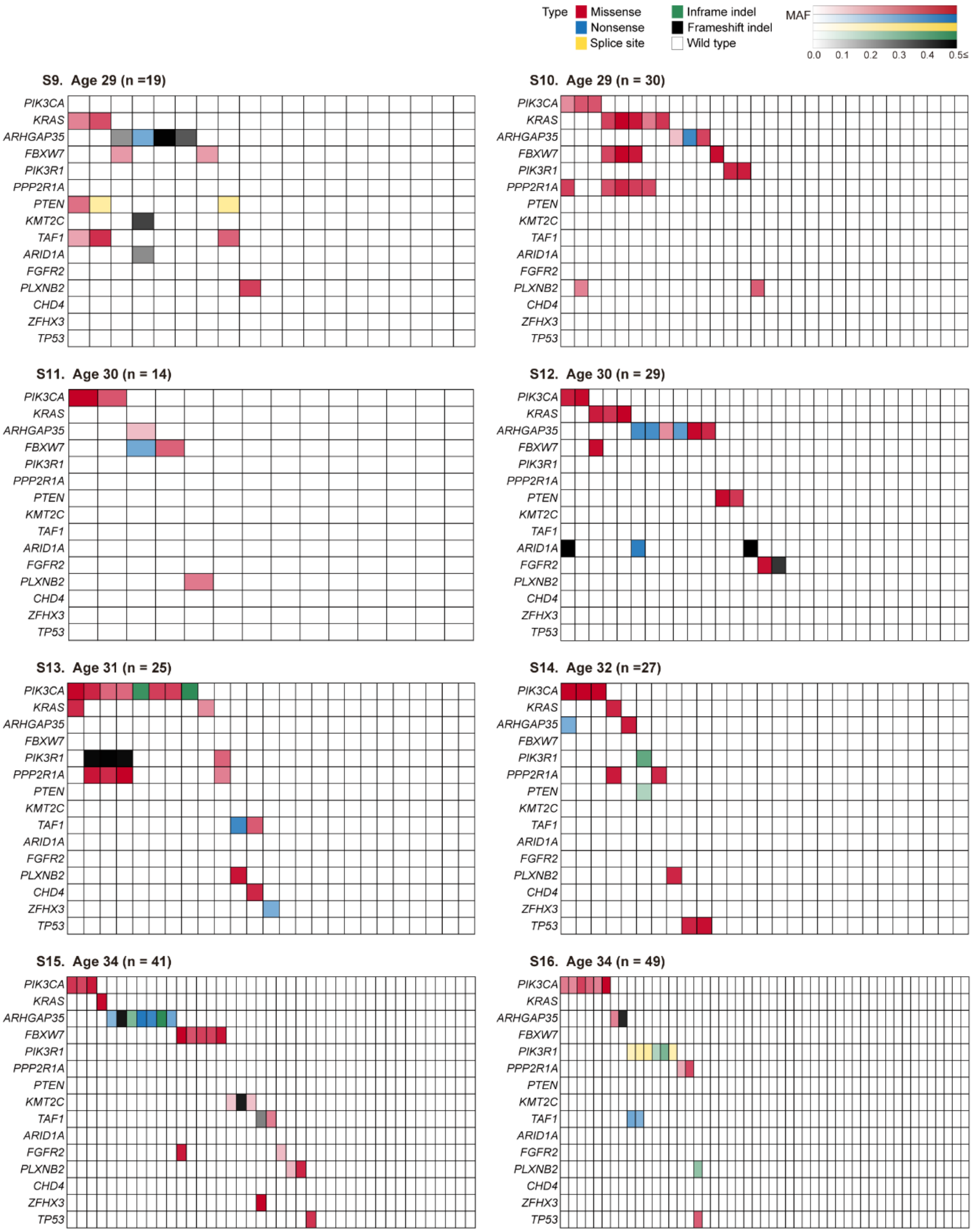
Type
Missense
Nonsense
Splice site
Inframe indel
Frameshift indel
Wild type
MAF
0.0 0.1 0.2 0.3 0.4 0.5≤
S9. Age 29 (n =19)
S10. Age 29 (n = 30)
S11. Age 30 (n = 14)
S12. Age 30 (n = 29)
S13. Age 31 (n = 25)
S14. Age 32 (n =27)
S15. Age 34 (n = 41)
S16. Age 34 (n = 49)
PIK3CA
KRAS
ARHGAP35
FBXW7
PIK3R1
PPP2R1A
PTEN
KMT2C
TAF1
ARID1A
FGFR2
PLXNB2
CHD4
ZFHX3
TP53

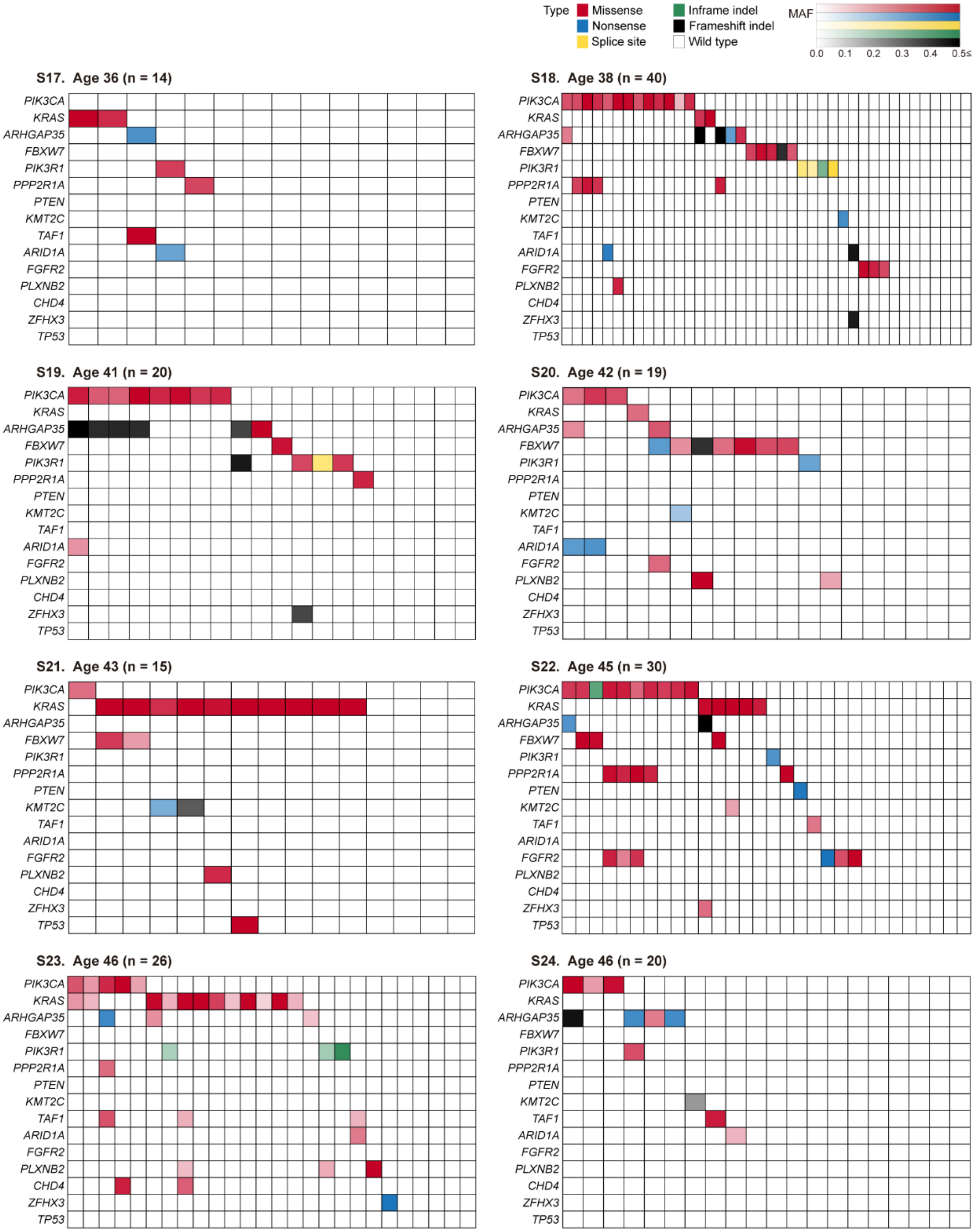

Type
Missense
Nonsense
Splice site
Inframe indel
Frameshift indel
Wild type
MAF
0.0
0.1
0.2
0.3
0.4
0.5≤
S17. Age 36 (n = 14)
S18. Age 38 (n = 40)
S19. Age 41 (n = 20)
S20. Age 42 (n = 19)
S21. Age 43 (n = 15)
S22. Age 45 (n = 30)
S23. Age 46 (n = 26)
S24. Age 46 (n = 20)
PIK3CA
KRAS
ARHGAP35
FBXW7
PIK3R1
PPP2R1A
PTEN
KMT2C
TAF1
ARID1A
FGFR2
PLXNB2
CHD4
ZFHX3
TP53

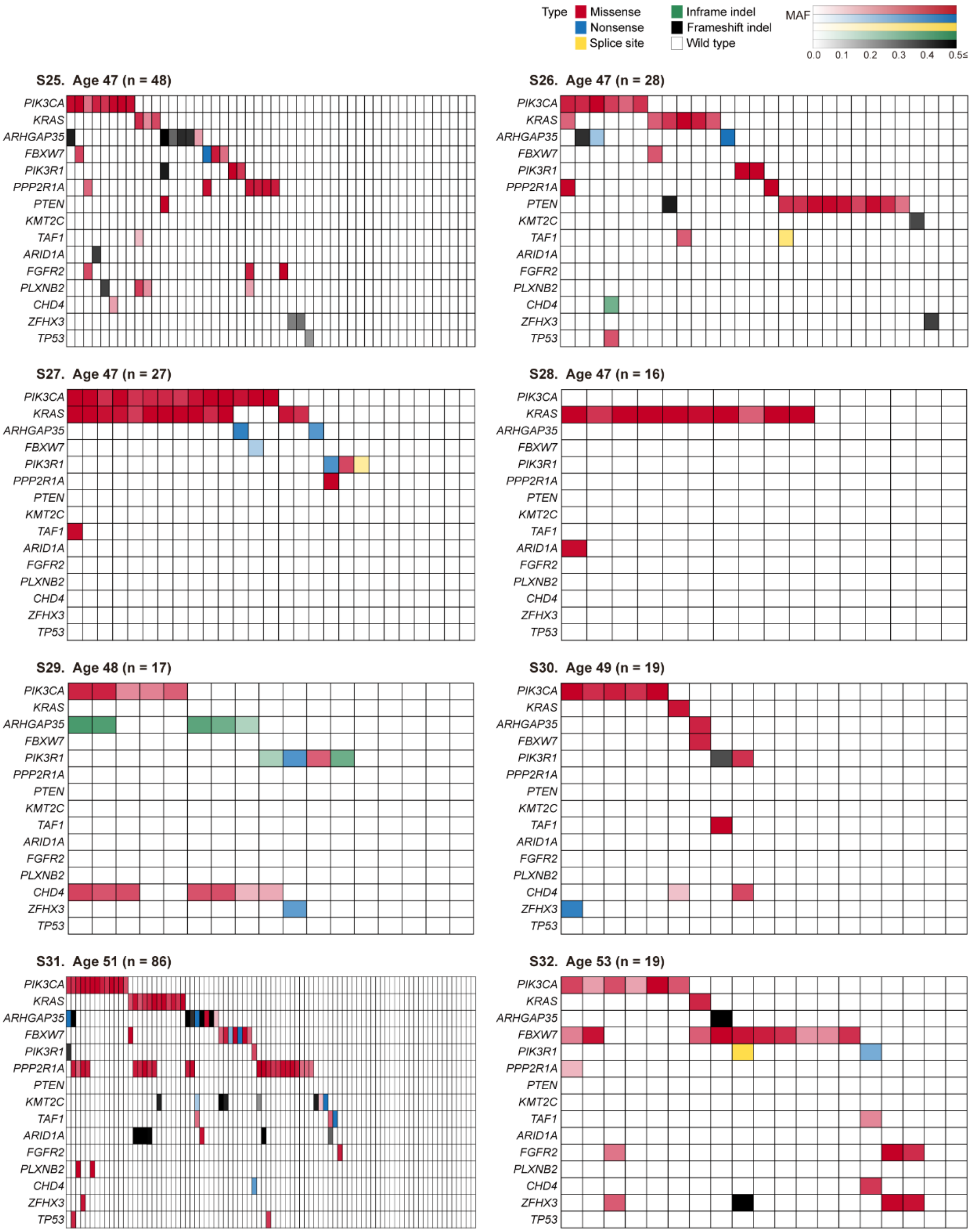


**Supplementary Figure 1. Heatmap of mutation profiling of the 15 most frequently mutated genes by subject sorted according to age. Related to Figure 1.**

Nonsilent mutations are color-coded: missense SNVs (red), nonsense SNVs (blue), splice-site SNVs (yellow), in-frame indels (green) and frameshifting indels (black). Color density indicates the MAF of each somatic mutation.

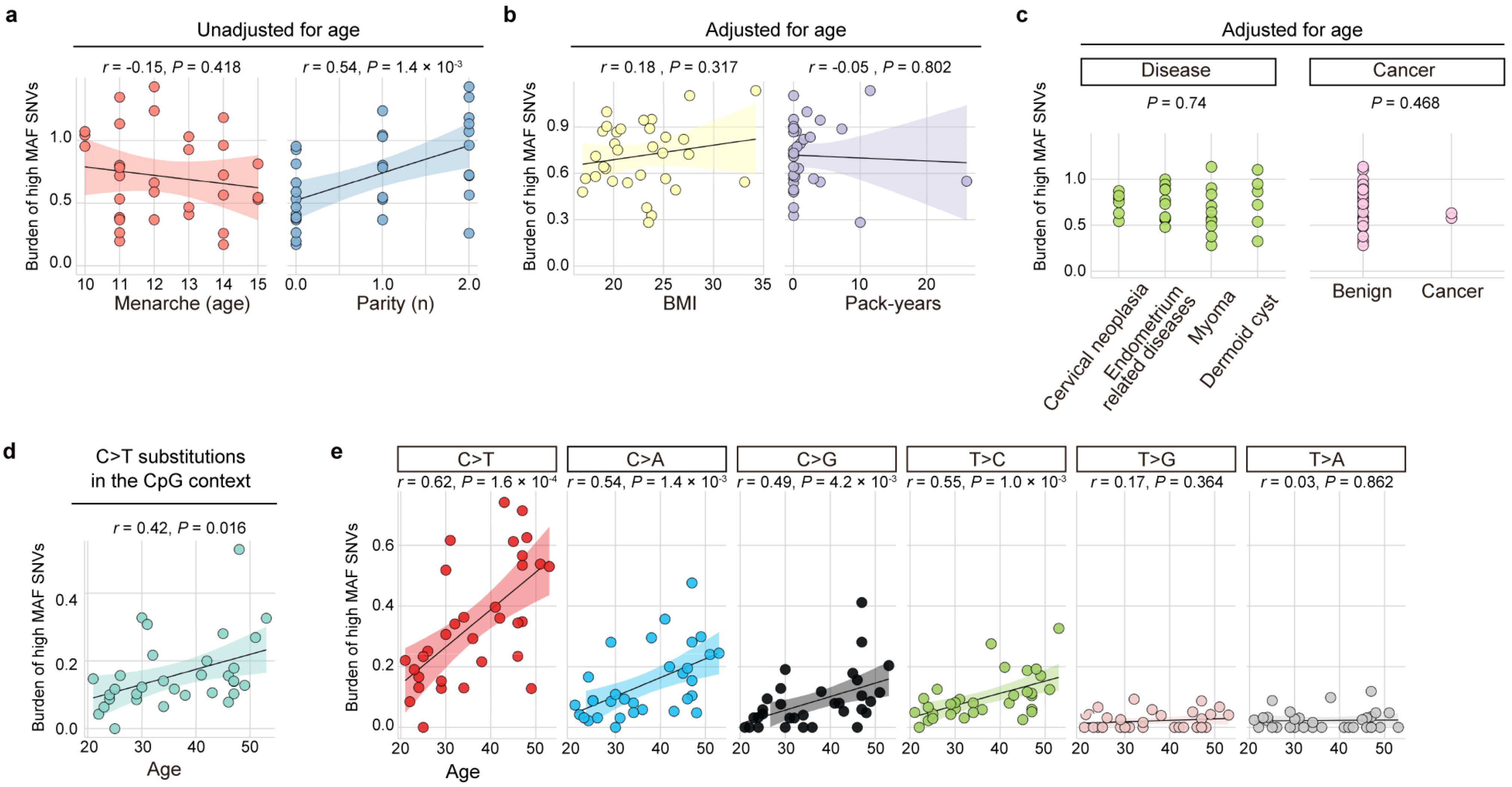


**Supplementary Figure 2. Correlations between the burdens of somatic SNVs with high MAF and clinical variables. Related to Figure 2.**

a) Linear relationships of age of menarche and number of parities with the burden of somatic SNVs with high MAF before adjustment for age. The number of parities showed a significant association due to the confounding effect of age. After adjustment for age, the association did not remain significant, as shown in Fig. 2c.

b) Linear relationships of BMI and pack-years with the burden of somatic SNVs with high MAF after adjustment for age.

c) Relationships of the affected status of gynecologic cancers and benign diseases with the burden of somatic SNVs with high MAF after adjustment for age (liner regression analysis with adjustment for age). The statistical significance level (*P*) based on one-way analysis of variance is shown. Unadjusted *P*-value is shown.

d) Linear relationships of age with the burden of C>T transitions at CpG motifs.

e) Linear relationships of age with burdens of the six pyrimidine substitutions.

a, b, d, e) Pearson's correlation coefficient ($r$) and the statistical significance level ($P$) based on two-tailed test are shown. Unadjusted $P$-value is shown. The slope of a linear regression line (solid line) with 95% CI (shaded area as error band) is plotted.

Source data are provided as a Source Data file.

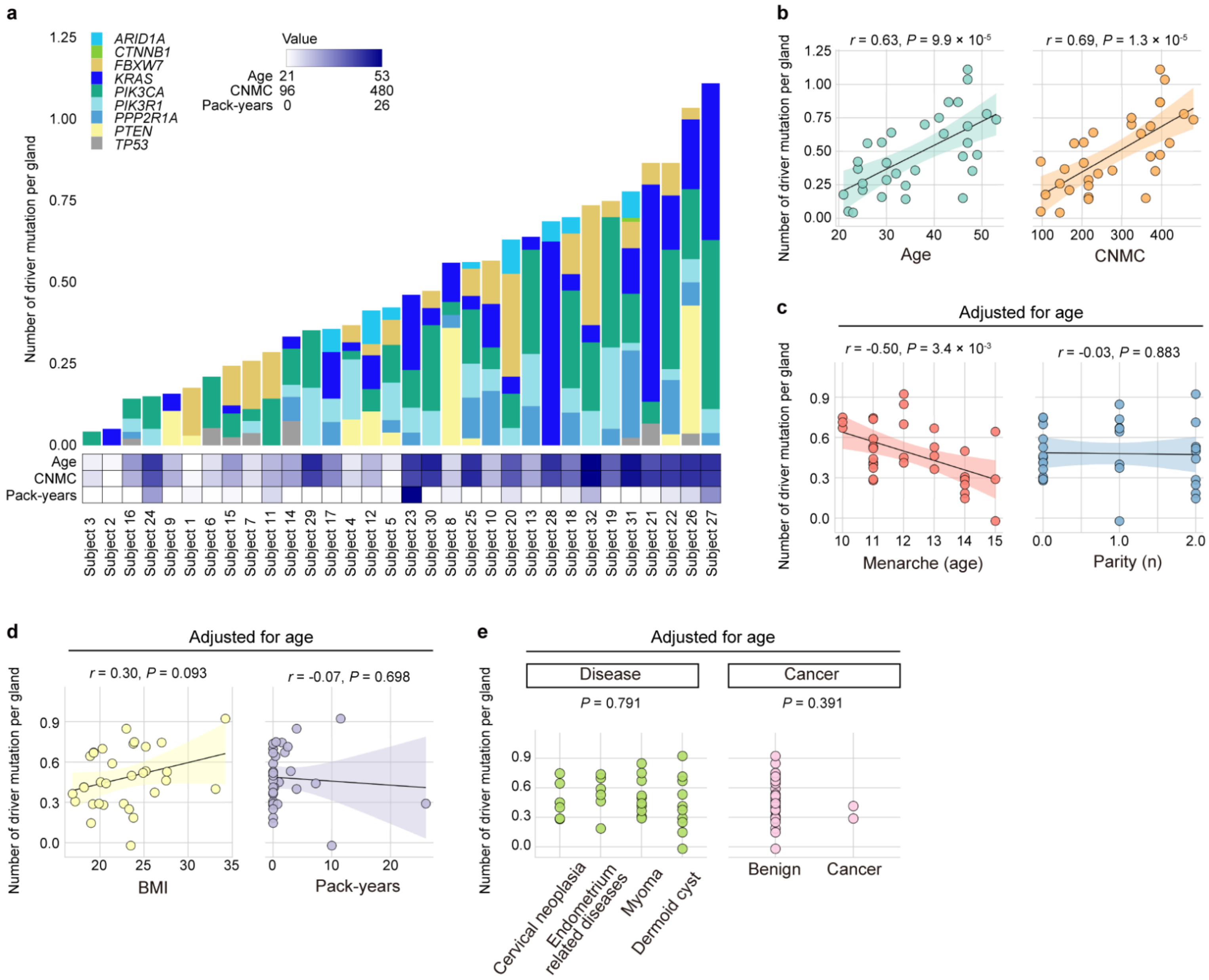


**Supplementary Figure 3. Correlations between the burden of mutations in cancer-driver genes and clinical variables. Related to Figure 2.**

a) Bar charts showing the burden of mutations in cancer-driver genes for 32 subjects stacked by the nine driver genes (*ARID1A*, *CTNNB1*, *FBXW7*, *KRAS*, *PIK3CA*, *PIK3R1*, *PPP2R1A*, *PTEN* and *TP53*). The burden is defined as the number of non-silent mutations in these nine genes per gland in each subject. Multiple glands from an individual subject are pooled. Subjects are sorted in ascending order according to burden of driver mutations. The heatmap below the bar charts represents age, CNMCs, and pack-years of cigarette smoking for the 32 subjects.

b) Linear relationships of age and CNMCs with the burden of driver mutations. The burden of driver mutations accumulates with age and CNMCs.

c) Linear relationships of age of menarche and number of parities with the burden of driver mutations after adjustment for age. The age of menarche showed a significant negative correlation.

d) Linear relationships of BMI and pack-years with the burden of driver mutations after adjustment for age.

b-d) Pearson's correlation coefficient (*r*) and the statistical significance level (*P*) based on two-tailed test are shown. Unadjusted *P*-value is shown. The slope of a linear regression line (solid line) with 95% CI (shaded area as error band) is plotted.

e) Relationships of the affected status of gynecologic cancers and benign diseases with the burden of driver mutations after adjustment for age (liner regression analysis with adjustment for age). The statistical significance level (*P*) based on one-way analysis of variance is shown. Unadjusted *P*-value is shown.

Source data are provided as a Source Data file.

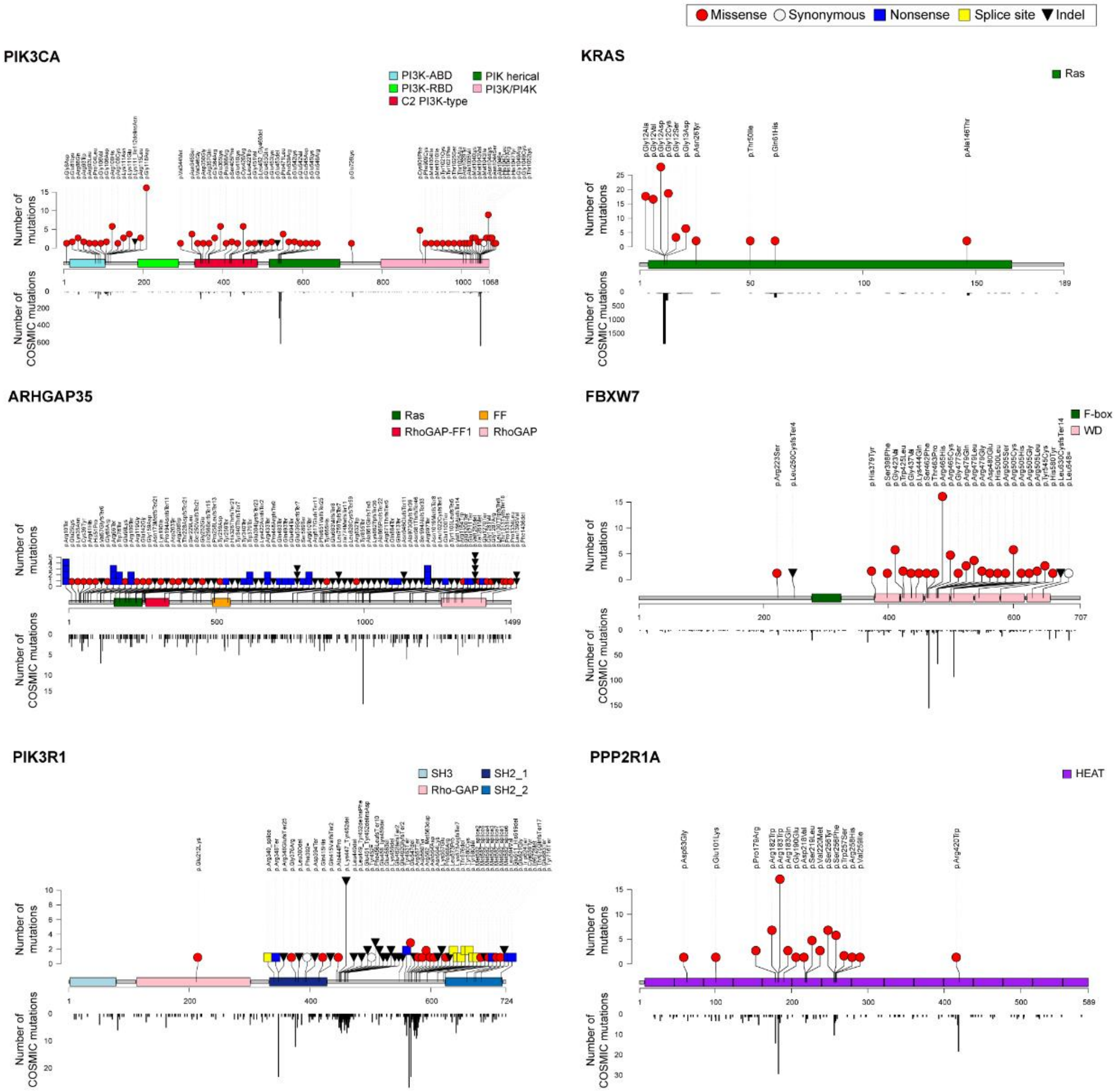

Missense
Synonymous
Nonsense
Splice site
Indel
PIK3CA
PI3K-ABD
PIK herical
PI3K-RBD
PI3K/PI4K
C2 PI3K-type
Number of mutations
Number of COSMIC mutations
KRAS
Ras
p.Gly12Ala
p.Gly12Val
p.Gly12Asp
p.Gly12Cys
p.Gly12Ser
p.Gly13Asp
p.Asn26Tyr
p.Thr50Ile
p.Gln61His
p.Ala146Thr
ARHGAP35
Ras
FF
RhoGAP-FF1
RhoGAP
FBXW7
F-box
WD
PIK3R1
SH3
SH2_1
Rho-GAP
SH2_2
PPP2R1A
HEAT
p.Asp63Gly
p.Glu101Lys
p.Pro179Arg
p.Arg182Trp
p.Arg183Trp
p.Arg183Gln
p.Gly190Glu
p.Asp218Val
p.Ser219Leu
p.Val220Met
p.Ser256Tyr
p.Ser256Phe
p.Trp257Ser
p.Arg258His
p.Val259Ile
p.Arg420Trp

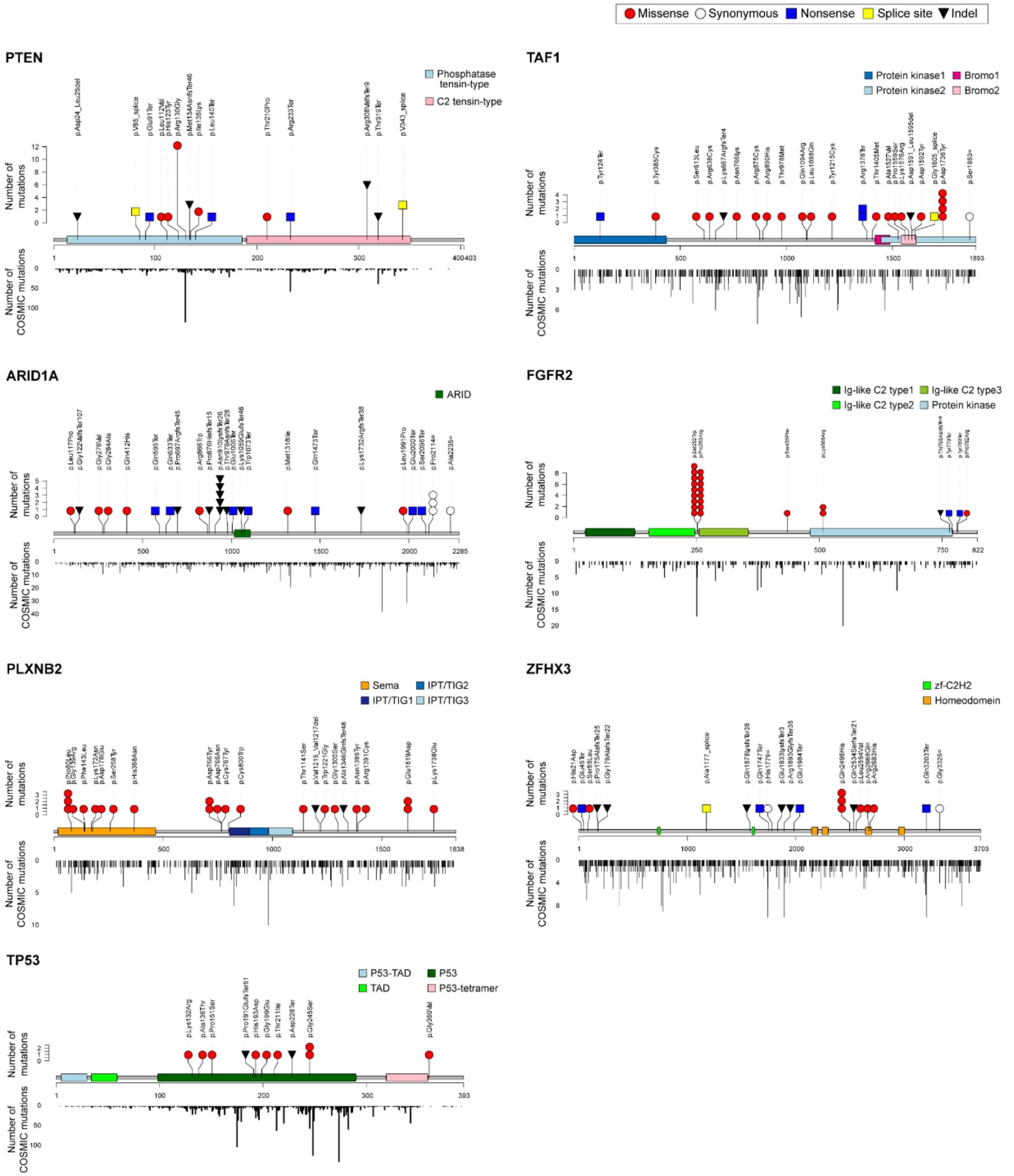


**Supplementary Figure 4. Lollipop plots for 13 genes. Related to Figure 3.**

Lollipop plots showing the locations of the identified somatic mutations in 13 genes along with known domain structures of the proteins encoded by these genes. Numbers refer to amino acid residues. The heights of the lollipops correspond to the number of mutations at each amino acid residue. Black bar charts indicate the number of somatic mutations deposited in the COSMIC database. The descriptions of mutations are located above the lollipops.

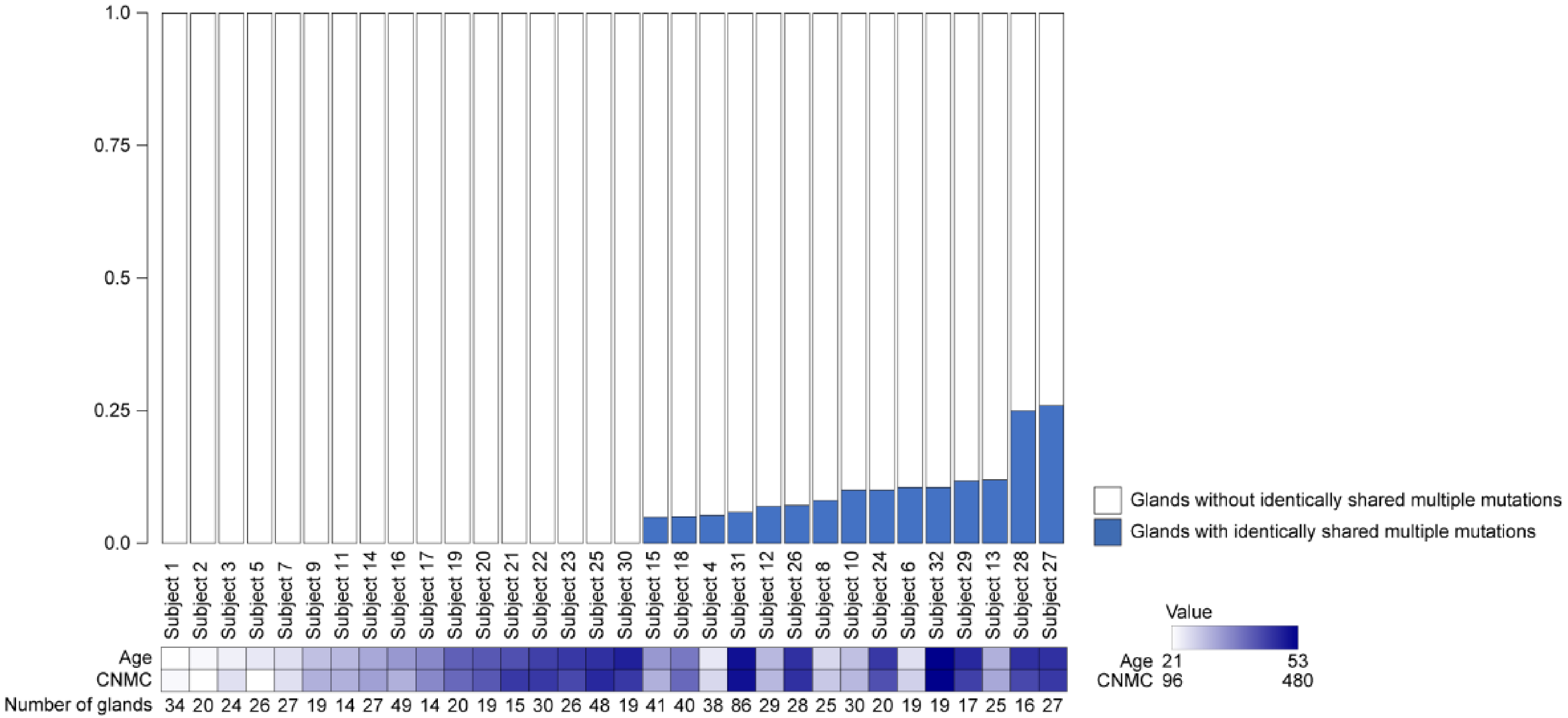


**Supplementary Figure 5. Proportions of glands with identically shared multiple mutations with high MAF. Related to Figure 3.**

Subjects are sorted in ascending order according to the proportions of glands with identically shared multiple mutations with high MAF (blue). The heatmap below the bar charts represents age and estimated CNMCs for the 32 subjects. Numbers below the heatmap show the total number of glands in each subject. Source data are provided as a Source Data file.

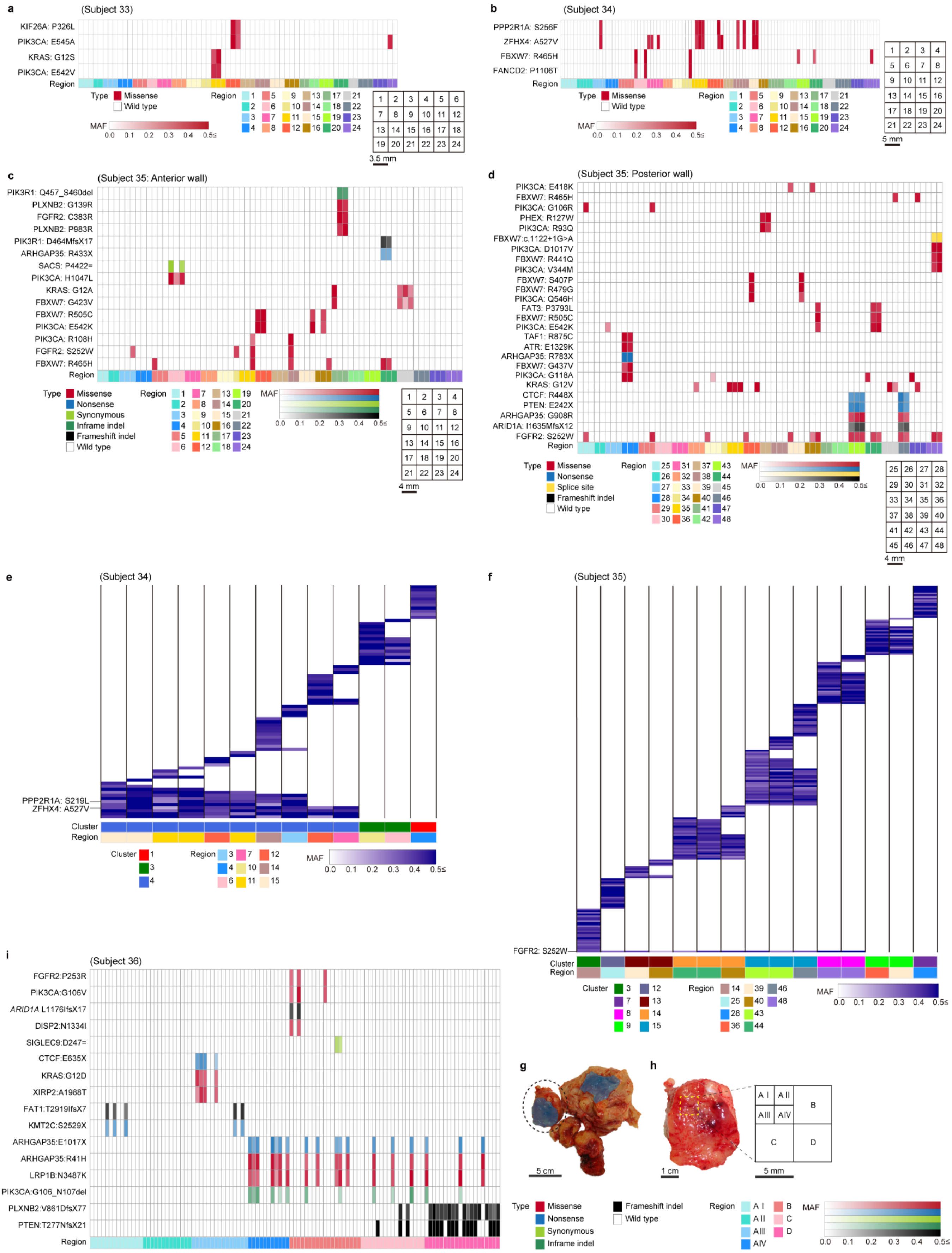

**Supplementary Figure 6. Landscape of somatic mutations in normal endometrial glands of subjects 33-36. Related to Figure 4 and 5.**

a-d) Heatmaps of the prevalence of somatic mutations based on target-gene sequencing in normal endometrial glands from subject 33 (a), subject 34 (b) and subject 35 (c and d).

e) Sharing pattern of somatic SNVs based on WES for subjects 34 (e) and 35 (f).

g) A macroscopic image of a tissue sample of the uterus obtained from a 50-year-old woman (subject 36), in which the endometrium is highlighted in light blue.

h) A magnified image of the endometrium circled by a dashed black line in panel (g). A part of the endometrium enclosed by the dashed yellow line was intensively analyzed. A schematic layout represents the partitioning of the resected endometrium into square grids with their identifiers.

i) Heatmap of the prevalence of somatic mutations based on target-gene sequencing for 109 normal endometrial glands from subject 36.

Mutations are color-coded: missense SNVs (red), nonsense SNVs (blue), splice-site SNVs (yellow), synonymous SNVs (light green), in-frame indels (green) and frameshifting indels (black). Color density indicates the MAF of each somatic mutation.

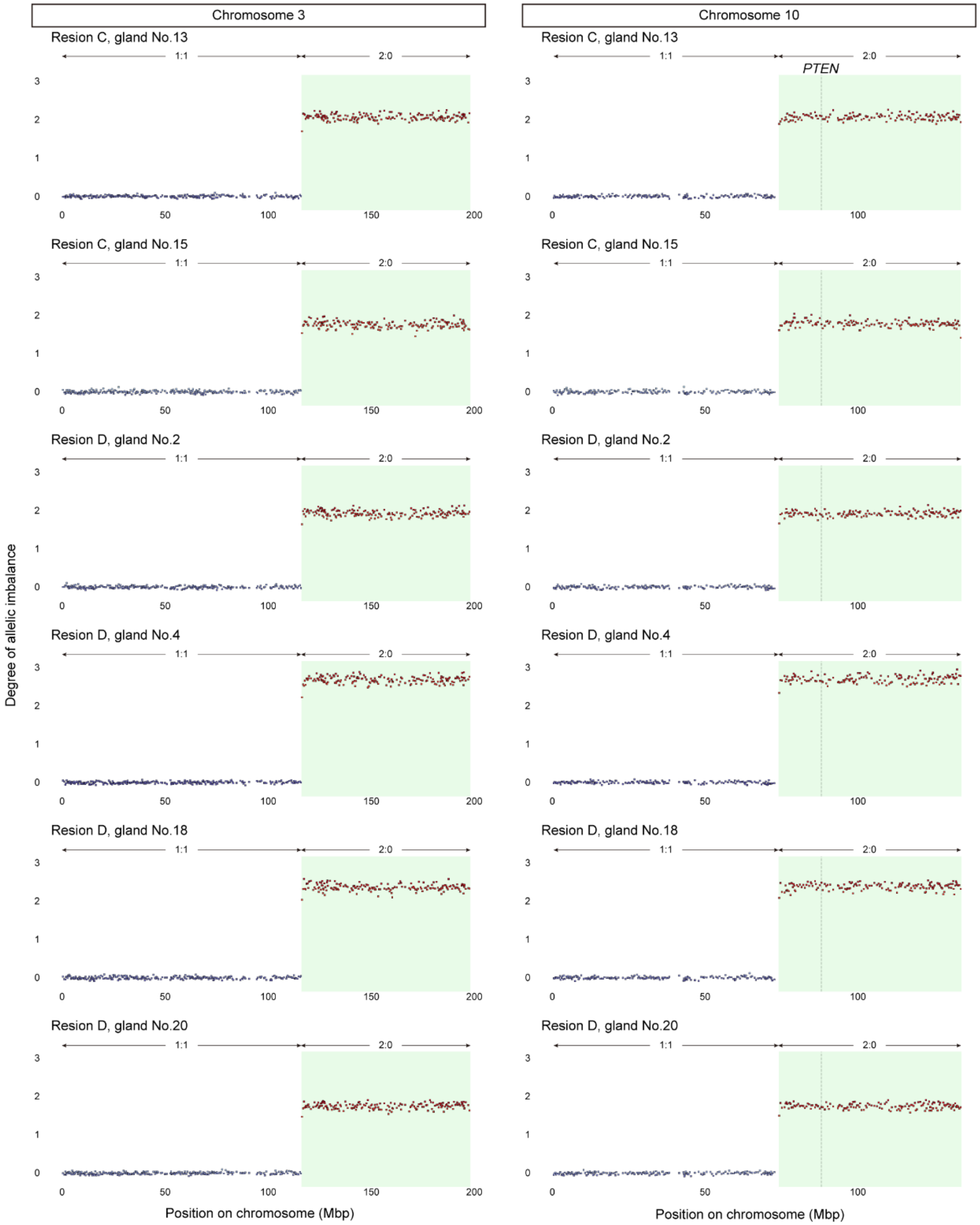


**Supplementary Figure 7. CN-LOHs at chromosomes 3 and 10 in six glands belonging to cluster C of subject 36. Related to Figure 5.**

Regions affected by CN-LOHs are highlighted in light green. The numbers separated by a colon are the major and minor copy numbers for the regions indicated by the arrows. Source data are provided as a Source Data file.

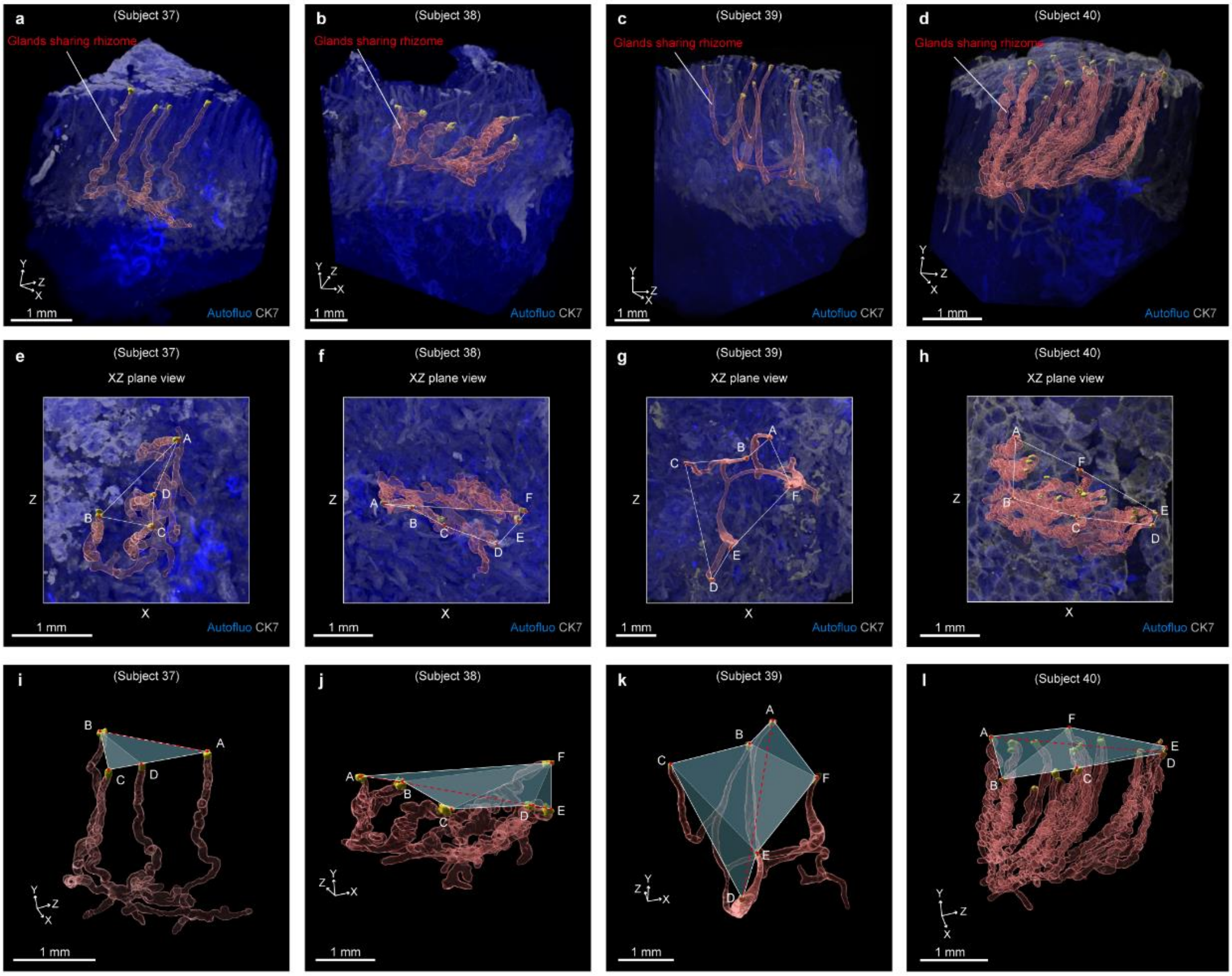


**Supplementary Figure 8. 3D imaging of normal proliferative endometrial tissues (subjects 36-39). Related to Figure 6.**

a-d). Reconstructed 3D image of endometrial tissue. Red objects: 3D structures of the glands sharing a rhizome. Yellow objects: tips of the glands sharing a rhizome.

e-h). XZ-plane-view images of glands sharing a rhizome in subjects 36-39. The tips of the glands used for the calculation of the longest distance and the area are highlighted.

i-l). Quantification of the longest distance between glands sharing a rhizome (red line) and the area occupied by the glands (light blue area).

3D images were obtained by light-sheet fluorescence microscopy. Autofluorescence and CK7-expressing endometrial epithelial cells were measured by excitation with 488 nm and 532 nm lasers, respectively. Red and yellow objects were made by the Surface module in Imaris software. Autofluo, autofluorescence; CK7, cytokeratin 7. Source data are provided as a Source Data file.

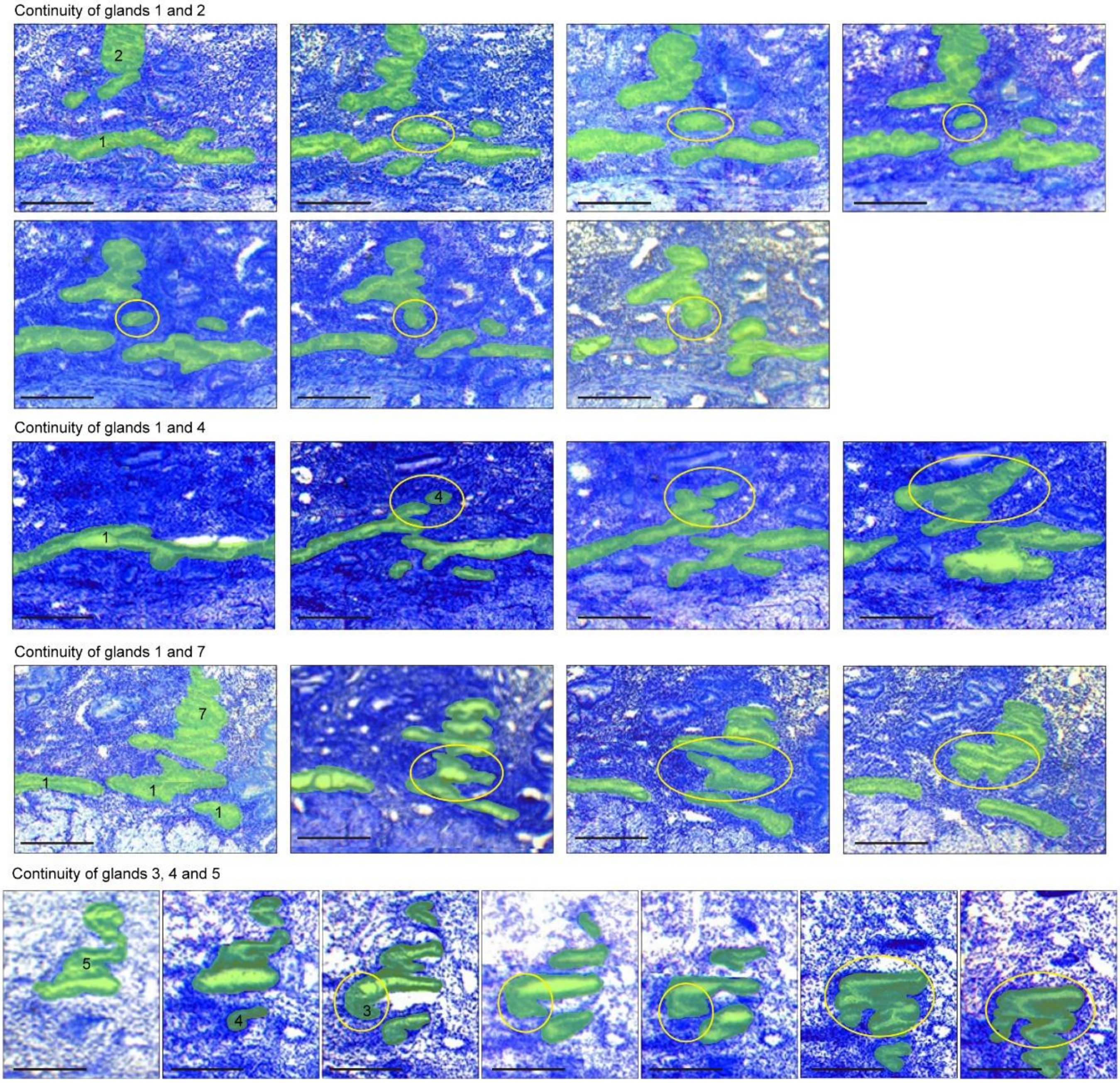
Continuity of glands 1 and 2
Continuity of glands 1 and 4
Continuity of glands 1 and 7
Continuity of glands 3, 4 and 5

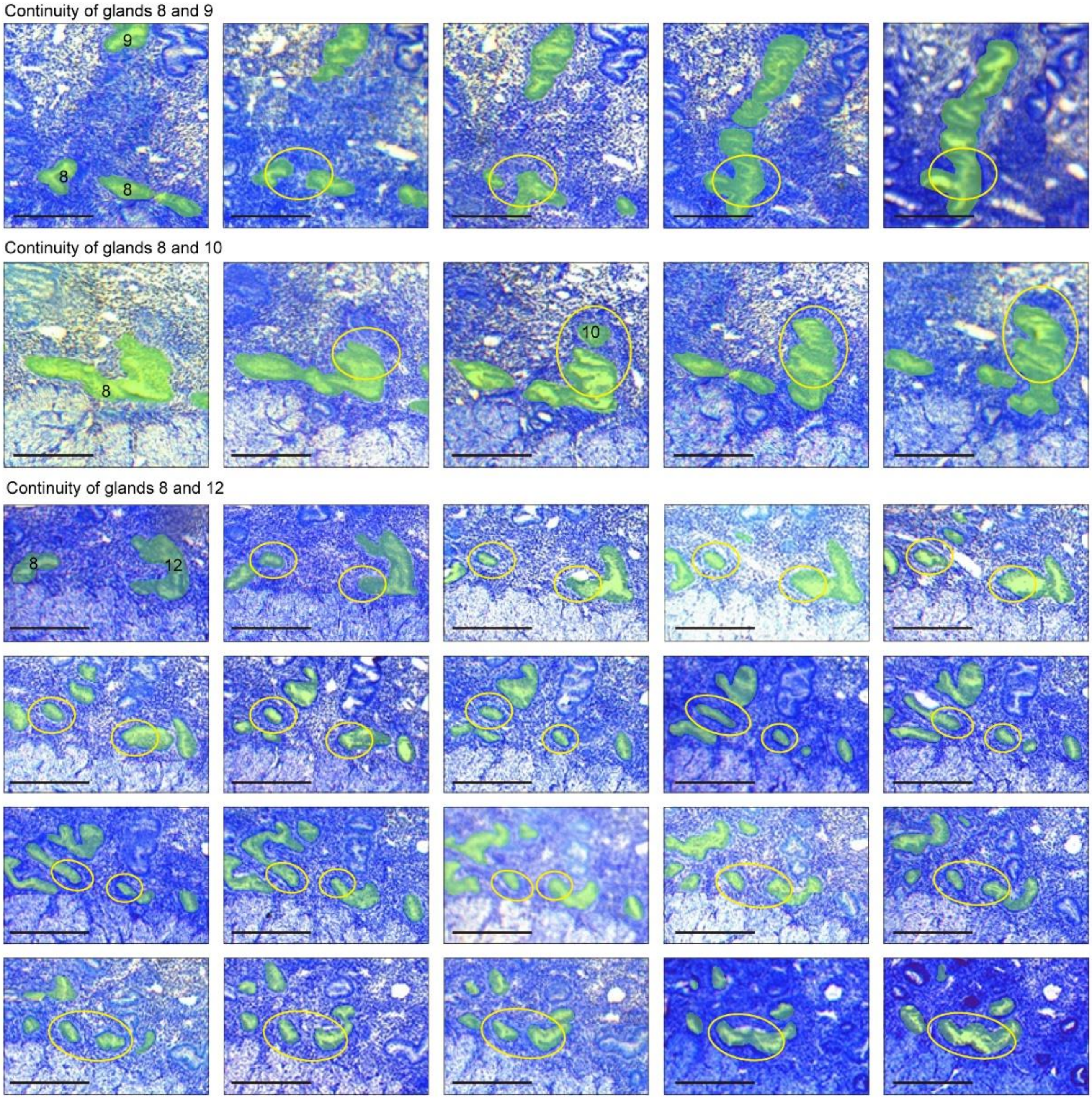


**Supplementary Figure 9. Evaluation of the continuity between glands based on serial sections of histology images before laser microdissection. Related to Figure 7.**

Toluidine blue-stained images with scale bar of 300 μm.

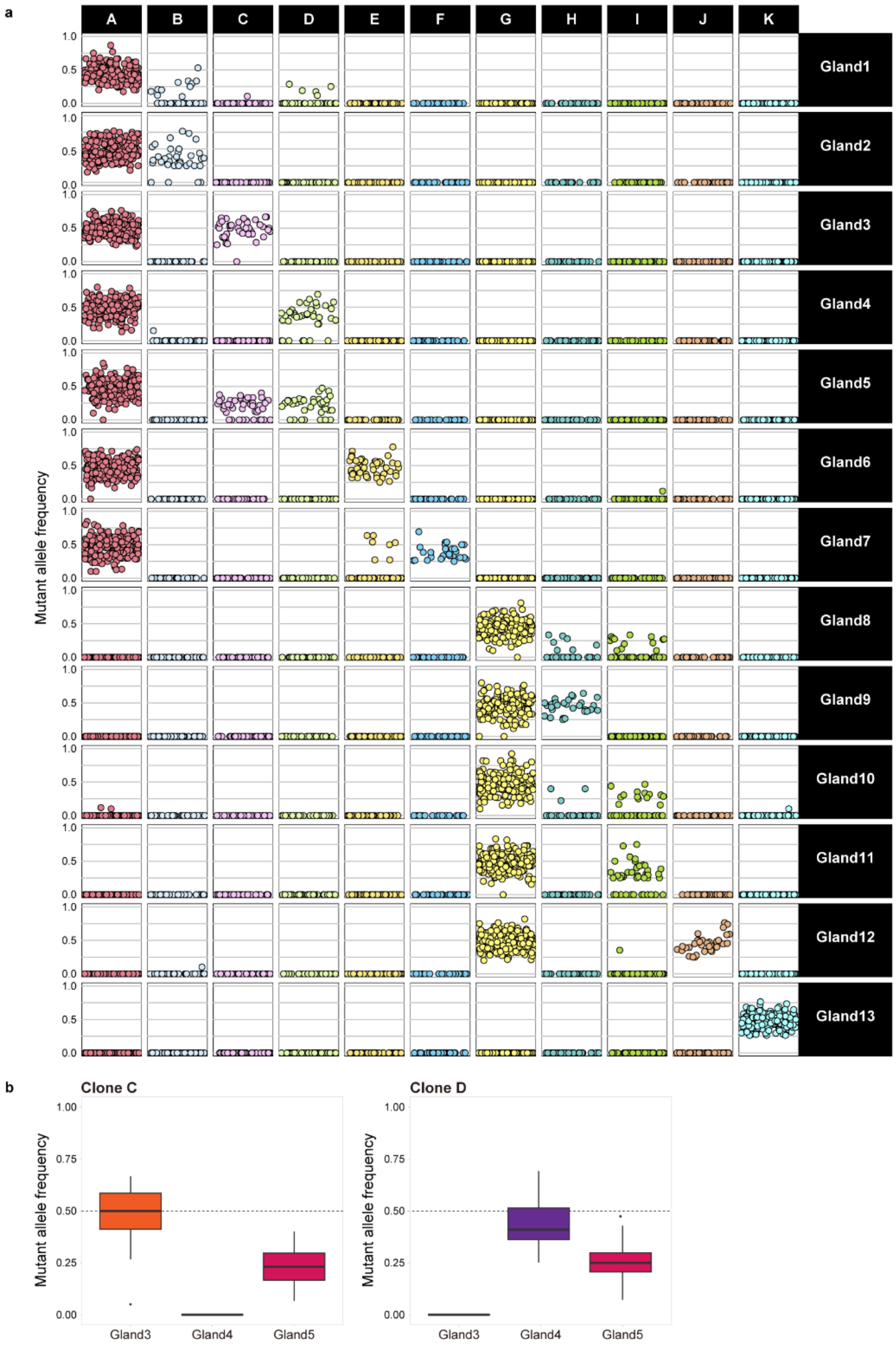


**Supplementary Figure 10. Clonal relationships among the 13 glands. Related to Figure 7.**

a) Somatic SNVs were classified into 11 clusters based on their MAF profiles with PyClone.

b) Distribution of the MAFs of somatic SNVs characterizing clones C and D in glands G3, G4 and G5. Box plots show the minima (bottom dot), the maxima (top dot), the median (middle line) and the first and third quartiles (boxes), whereas the whiskers show 1.5× the interquartile range (IQR). The numbers of mutations (*n*) shared uniquely in clones C and D are 35 and 22, respectively.

**Supplementary Table 1. Clinical characteristics of 32 subjects of varying ages for target-gene sequencing, related to Figure 1.**

| Subject | Age | Clinical diagnosis | Menstrual cycle (day) | Menarche (age) | Parity | CNMC | Body mass index | Pack-years |
|---|---|---|---|---|---|---|---|---|
| 1 | 21 | CIN3/CIS | 19 | 12 | 0 | 108 | 20.1 | 1 |
| 2 | 22 | Endometriosis | 32 | 14 | 0 | 96 | 17.2 | 0 |
| 3 | 23 | CIN3/CIS | 22 | 11 | 0 | 144 | 20.4 | 0 |
| 4 | 24 | Ovarian dermoid cyst | 25 | 11 | 0 | 156 | 21.4 | 0 |
| 5 | 24 | CIN3/CIS | 7 | 15 | 1 | 96 | 18.9 | 0.4 |
| 6 | 25 | Ovarian clear cell carcinoma | 14 | 11 | 0 | 168 | 18.2 | 0.2 |
| 7 | 25 | Ovarian dermoid cyst | 23 | 13 | 0 | 144 | 27.5 | 0 |
| 8 | 26 | CIN3/CIS | 18 | 11 | 0 | 180 | 27.0 | 1.5 |
| 9 | 29 | Endometriosis | 22 | 11 | 0 | 216 | 22.7 | 0 |
| 10 | 29 | Ovarian dermoid cyst | 22 | 12 | 0 | 204 | 20.3 | 0 |
| 11 | 30 | CIN3/CIS | 11 | 11 | 1 | 216 | 33.1 | 4 |
| 12 | 30 | Ovarian dermoid cyst | 29 | 13 | 0 | 204 | 27.6 | 0 |
| 13 | 31 | Ovarian dermoid cyst | 25 | 11 | 1 | 228 | 23.8 | 0 |
| 14 | 32 | Myoma uteri, Ovarian dermoid cyst | 17 | 12 | 0 | 240 | 18.2 | 0 |
| 15 | 34 | Cervical adenocarcinoma | unknown | 14 | 2 | 216 | 19.2 | 0.75 |
| 16 | 34 | Ovarian dermoid cyst | 25 | 14 | 2 | 216 | 23.8 | 0 |
| 17 | 36 | Endometriosis, Myoma uteri | 9 | 13 | 0 | 276 | 16.9 | 0 |
| 18 | 38 | Endometriosis | 13 | 10 | 1 | 324 | 19.3 | 2 |
| 19 | 41 | Myoma uteri | 10 | 13 | 1 | 324 | 19.3 | 0 |
| 20 | 42 | Myoma uteri | 1 | 11 | 2 | 348 | 25.2 | 3 |
| 21 | 43 | Endometriosis | 13 | 10 | 0 | 396 | 23.9 | 0.5 |
| 22 | 45 | Myoma uteri | 23 | 10 | 2 | 396 | 25.2 | 2.5 |
| 23 | 46 | Myoma uteri | 27 | 15 | 0 | 372 | 19.9 | 26 |
| 24 | 46 | Myoma uteri | 18 | 15 | 1 | 360 | 23.5 | 10 |
| 25 | 47 | Myoma uteri | 19 | 11 | 1 | 420 | 26.2 | 0 |
| 26 | 47 | Adenomyosis | 63 | 12 | 1 | 408 | 23.0 | 4 |
| 27 | 47 | Myoma uteri | 2 | 12 | 2 | 396 | 34.2 | 11.5 |
| 28 | 47 | Endometriosis | 11 | 14 | 2 | 372 | 23.6 | 0 |
| 29 | 48 | Myoma uteri | 23 | 14 | 2 | 384 | 19.0 | 0 |
| 30 | 49 | Myoma uteri | 14 | 14 | 2 | 396 | 23.3 | 0 |
| 31 | 51 | Endometriosis | unknown | 11 | 2 | 456 | 24.9 | 0 |
| 32 | 53 | Adenomyosis | 25 | 11 | 2 | 480 | 20.7 | 7.25 |

CNMC (cumulative number of menstrual cycles) was calculated as below: (age – menarche age) × 12 – parity × 12.

Pack-years was calculated as below: (number of cigarettes smoked per day/20) × number of years smoked.

**Supplementary Table 2. Clinical characteristics of four subjects who underwent spatially resolved target-gene sequencing, related to Figures 4 and 5.**

| Subject | Age | Clinical diagnosis | Menstrual cycle (day) | Menarche (age) | Parity | CNMC | Body mass index | Pack-years | No. of grids | No. of picked up single endometrial glands per segment |
|---|---|---|---|---|---|---|---|---|---|---|
| 33 | 38 | Endometriosis | 5 | 12 | 1 | 300 | 21.6 | 0 | 24 | 3 |
| 34 | 41 | Myoma uteri | 12 | 12 | 3 | 312 | 27.2 | 1.25 | 24 | 5 |
| 35 | 46 | CIS | 24 | 12 | 0 | 408 | 21.9 | 0 | 48 | 3 |
| 36 | 50 | Myoma uteri | 28 | 14 | 1 | 420 | 24.2 | 0 | 7 | 12-20 |

CNMC (cumulative number of menstrual cycles) was calculated as below: (age – menarche age) × 12 – parity × 12.

Pack-years was calculated as below: (number of cigarettes smoked per day/20) × number of years smoked.

**Supplementary Table 3. Clinical characteristics of subjects in 3D imaging analysis of glands sharing a rhizome, related to Figure 6.**

| Subject | Age | Clinical diagnosis | Menstrual cycle | Parity | Body mass index | Longest distance between glands sharing the rhizome (mm) | Area occupied by glands sharing the rhizome ($mm^2$) |
|---|---|---|---|---|---|---|---|
| 37 | 30 | Cervical squamous cell carcinoma | Proliferative phase | 1 | 34.1 | 1.3 | 0.3 |
| 38 | 39 | Cervical squamous cell carcinoma | Proliferative phase | 2 | 17.9 | 3.3 | 2.0 |
| 39 | 46 | Myoma uteri | Proliferative phase | 0 | 25.0 | 3.8 | 4.7 |
| 40 | 52 | Myoma uteri | Proliferative phase | 3 | 24.0 | 2.8 | 1.8 |
