# Supplementary material for: Spatiotemporal dynamics of clonal selection and diversification in normal endometrial epithelium
Source: Nat Commun. 2022 Feb 17;13:943. doi: 10.1038/s41467-022-28568-2 (PMC8854701; doi:10.1038/s41467-022-28568-2)
Supplement: Supplementary file 7 — Source data [file 41467_2022_28568_MOESM7_ESM.zip › source_data.docx]

For each source data file, its relationship with figures in the paper is described. The information included in each source data file is shown as the following form: column number, heading: description.

source_data_01.xlsx, related to Fig. 1c

The data used to generate a bar plot for proportions of glands with somatic mutations in 15 genes

1, gene: name of a frequently mutated gene

2, SNV: proportion of glands with SNV in the respective gene

3, indel: proportion of glands with indel in the respective gene

4, both: proportion of glands with both SNV and indel in the respective gene

source_data_02.xlsx, related to Fig. 1d

The data used to generate box plots for mutant allele frequencies of somatic mutations in 15 genes

1, gene: name of a gene affected by a somatic mutation

2, mutant_allele_frequency: mutant allele frequency of a somatic mutation

3, effect: effect of a somatic mutation on the respective gene

4, variant_type: type of a somatic mutation (SNV or indel)

source_data_03.xlsx, related to Fig. 1e

The data used to generate a density plot for mutant allele frequencies

1, effect: effect of a somatic mutation

2, mutation_allele_frequency: mutant allele frequency of a somatic mutation

source_data_04.xlsx, related to Fig. 1f

The data used to generate a bar plot for mutation spectrum

1, substitution: type of substitution using the pyrimidine base

2, trinucleotide: type of trinucleotide context

3, context_lego: combination of substitution and trinucleotide context for lego plot

4, context_sigfit: combination of substitution and trinucleotide context for sigfit analysis

5, freq_high_maf_SNV: frequency of somatic SNVs in the respective type of trinucleotides context

source_data_05.xlsx, related to Fig. 1g

The data used to generate a bar plot for the contributions of mutational signatures

1, signature: name of a COSMIC SBS signature

2, value: estimated contribution of the SBS signature

3, lower_90: lower 90% HPD interval for the estimated contribution

4, upper_90: upper 90% HPD interval for the estimated contribution

source_data_06.xlsx, related to Fig. 2a,e and Supplementary Fig. 2e

The data used to generate a piled bar plot for mutational burdens

1, subject: identifier of a subject

2, burden_C>A: burden of C>A substitutions in the subject

3, burden_C>G: burden of C>G substitutions in the subject

4, burden_C>T: burden of C>T substitutions in the subject

5, burden_T>A: burden of T>A substitutions in the subject

6, burden_T>C: burden of T>C substitutions in the subject

7, burden_T>G: burden of T>G substitutions in the subject

8, age: age of the subject

9, cum_num_menstrual_cycles: estimated cumulative number of menstrual cycles of the subject

10, pack_years: pack years of smoking of the subject

source_data_07.xlsx, related to Fig. 2b and Supplementary Fig. 2a

The data used to generate scatter plots showing relationships between mutational burden and clinical variables

1, subject: identifier of a subject

2, burden: burden of somatic SNVs with high mutant allele frequencies in the subject

3, age: age of the subject

4, cum_num_menstrual_cycles: estimated cumulative number of menstrual cycles of the subject

5, menarche: age of menarche of the subject

6, parity: parity of the subject

source_data_08.xlsx, related to Fig. 2c, Supplementary Fig. 2b and c

The data used to generate scatter plots showing relationships between age-adjusted mutational burden and clinical variables

1, subject: identifier of a subject

2, burden_age_adjusted: burden of somatic SNVs with high mutant allele frequencies after the adjustment for the effect of age in the subject

3, menarche: age of menarche of the subject

4, parity: parity of the subject

5, BMI: body mass index

6, pack_years: pack years of smoking of the subject

7, disease: disease status of the subject (cervical neoplasia, dermoid cyst, endometrium-related disease, or myoma)

8: cancer: cancer status of the subject (1, presence; or 0, absence)

source_data_09.xlsx, related to Fig. 2d and Supplementary Fig. 2d

1, subject: identifier of a subject

2, burden_5_methylcytosine: burden of somatic C>T substitutions with high mutant allele frequencies in the NpCpG context in the subject

3, age: age of the subject

4, cum_num_menstrual_cycles: estimated cumulative number of menstrual cycles of the subject

source_data_10.xlsx, related to Supplementary Fig. 3a

1, subject: identifier of a subject

2, age: age of the subject

3, cum_num_menstrual_cycles: estimated cumulative number of menstrual cycles of the subject

4, pack_years: pack years of smoking of the subject

5, ARID1A_burden: burden of somatic mutations with high mutant allele frequencies in ARID1A in the subject

6, CTNNB1_burden: burden of somatic mutations with high mutant allele frequencies in CTNNB1 in the subject

7, FBXW7_burden: burden of somatic mutations with high mutant allele frequencies in FBXW7 in the subject

8, KRAS_burden: burden of somatic mutations with high mutant allele frequencies in KRAS in the subject

9, PIK3CA_burden: burden of somatic mutations with high mutant allele frequencies in PIK3CA in the subject

10, PIK3R1_burden: burden of somatic mutations with high mutant allele frequencies in PIK3R1 in the subject

11, PPP2R1A_burden: burden of somatic mutations with high mutant allele frequencies in PPP2R1A in the subject

12, PTEN_burden: burden of somatic mutations with high mutant allele frequencies in PTEN in the subject

13, TP53_burden: burden of somatic mutations with high mutant allele frequencies in TP53 in the subject

source_data_11.xlsx, related to Supplementary Fig. 3b

1, subject: identifier of a subject

2, age: age of the subject

3, cum_num_menstrual_cycles: estimated cumulative number of menstrual cycles of the subject

4, driver_mutation_burden: burden of somatic mutations with high mutant allele frequencies in ARID1A, CTNNB1, FBXW7, KRAS, PIK3CA, PIK3R1, PPP2R1A, PTEN and TP53 in the subject

source_data_12.xlsx, related to Supplementary Fig. 3c,d and e

1, subject: identifier of a subject

2, parity: parity of the subject

3, menarche: age of menarche of the subject

4, BMI: body mass index

5, pack_years: pack years of smoking of the subject

6, disease: disease status of the subject (cervical neoplasia, dermoid cyst, endometrium-related disease, or myoma)

7: cancer: cancer status of the subject (1, presence; or 0, absence)

8, driver_mutation_burden_age_adjusted: burden of somatic mutations with high mutant allele frequencies in ARID1A, CTNNB1, FBXW7, KRAS, PIK3CA, PIK3R1, PPP2R1A, PTEN and TP53 after the adjustment for the effect of age in the subject

source_data_13.xlsx, related to Fig. 3g

1, analysis: four analyses including different sets of genes (“all_genes”, “cancer_gene_census”, “pan_gynecologic_cancer_genes”, and “non_cancer_genes”)

2, type: two types of analyses targeting missense or nonsense mutations

3, num_nonsynonymous_mut: the observed number of nonsynonymous mutations in endometrial glands
4, num_synonymous_mut: the observed number of synonymous mutations in endometrial glands
5, num_nonsynonymous_mut_exp: the expected number of nonsynonymous mutations
6, num_synonymous_mut_exp: the expected number of synonymous mutations

7, dNdS_value: estimated dN/dS value

8, lower_95: lower 95% confidence interval for the estimated dN/dS value

9, upper_95: upper 95% confidence interval for the estimated dN/dS value

10, p_value: P value for the significance of the estimated dN/dS value

source_data_14.xlsx, related to Fig. 3h and i

1, gene: name of gene

2, dN_missense: estimated dN value for missense mutations in the gene

3, dN_nonsense: estimated dN value for nonsense mutations in the gene

4, dS: estimated dS value for synonymous mutations in the gene

5, role: role of the gene: TSG (tumor suppressor gene), oncogene, or unknown

source_data_15.xlsx, related to Supplementary Fig. 5

1, subject: identifier of a subject

2, age: age of the subject

3, cum_num_menstrual_cycles: estimated cumulative number of menstrual cycles of the subject

4, num_glands: number of glands analyzed for the subject

5, num_glands_with_identically_shared_mutations: number of glands that had multiple identical mutations with other glands within the subject

6, prop_glands_with_identically_shared_mutations: proportion of glands that had multiple identical mutations with other glands within the subject

source_data_16.xlsx, related to Fig. 5f and Supplementary Fig. 7

1, sample: identifier for a gland in a subject

2, chr: chromosome of a germline variant

3, pos: position of a germline variant

4, norm_log_or: normalized log odds ratio of the variant allele read count in the gland and blood pair

source_data_17.xlsx, related to Fig. 5g

1, cluster: identifier for cluster of glands that were determined based on shared mutation status

2, sample: identifier for a gland in a subject

3, num_public: number of public mutations

4, num_partially_shared: number of partially shared mutations

5, num_private: number of private mutations

6, prop_public: proportion of public mutations

7, prop_partially_shared: proportion of partially shared mutations

8, prop_private: proportion of private mutations

source_data_18.xlsx, related to Fig. 5h

1, mut_group: sharing status of a mutation among glands in a cluster

2, sample: identifier for a gland in a subject

3, chr: chromosome of a mutation

4, pos: position of a mutation

5, ref: reference allele of a mutation

6, alt: alternative allele of a mutation

7, purity: purity of a clone with copy-neutral loss-of-heterozygosity in a gland

8, num_ref_read: number of reads with reference allele at the mutation site

9: num_alt_read: number of reads with mutant allele at the mutation site

10: depth: number of depth at the mutation site

11, mutant_allele_frequency: mutant allele frequency of a mutation

source_data_19.xlsx, related to Fig. 5i

1, chromosome: chromosome of a CN-LOH event

2, mutation_group: before or after the CN-LOH event

3, mutation_burden: mutation burden before or after the CN-LOH event

4, prop_mutation_burden: proportion of mutation burden before or after the CN-LOH event

source_data_20.xlsx, related to Fig. 6d and Supplementary Fig. 8

1, name: Identifier of the tip of the subject's gland

2, position X (um): X-axis coordinates of the tip

3, position Y (um): Y-axis coordinates of the tip

4, position Z (um): Z-axis coordinates of the tip

source_data_21.xlsx, related to Fig. 7i

1, group: identifier for group of glands that were linked by rhizome structures

2, sample: identifier for a gland in a subject

3, num_public: number of public mutations

4, num_partially_shared: number of partially shared mutations

5, num_private: number of private mutations

6, prop_public: proportion of public mutations

7, prop_partially_shared: proportion of partially shared mutations

8, prop_private: proportion of private mutations

source_data_22.xlsx, related to Supplementary Fig. 10a

1, chr: chromosome of a mutation

2, pos: position of the mutation

3, ref: reference allele

4, alt: alternative allele

5, cluster: cluster into which the mutation is grouped

6, S41_G1: mutant_allele_frequency in S41_G1

7, S41_G2: mutant_allele_frequency in S41_G2

8, S41_G3: mutant_allele_frequency in S41_G3

9, S41_G4: mutant_allele_frequency in S41_G4

10, S41_G5: mutant_allele_frequency in S41_G5

11, S41_G6: mutant_allele_frequency in S41_G6

12, S41_G7: mutant_allele_frequency in S41_G7

13, S41_G8: mutant_allele_frequency in S41_G8

14, S41_G9: mutant_allele_frequency in S41_G9

15, S41_G10: : mutant_allele_frequency in S41_G10

16, S41_G11: mutant_allele_frequency in S41_G11

17, S41_G12: mutant_allele_frequency in S41_G12

18, S41_G13: mutant_allele_frequency in S41_G13

source_data_23.xlsx, related to Supplementary Fig. 10b

1, type: whether a mutation is shared uniquely in clones C or D (denoted as “shared_3_5” or “shared_4_5”, respectively)

2, chr: chromosome of the mutation

3, pos: position of the mutation

4, ref: reference allele

5, alt: alternative allele

6, S41-G3: mutant allele frequency in S41-G3

7, S41-G4: mutant allele frequency in S41-G4

8, S41-G5: mutant allele frequency in S41-G5
